# Effects of pupillary dilation on ocular optical biometry outcomes in pediatric patients

**DOI:** 10.5935/0004-2749.20200041

**Published:** 2020

**Authors:** Selahattin Balsak

**Affiliations:** 1 Department of Ophthalmology, Diyarbakır Gazi Yaşargil Training and Research Hospital, Diyarbakır, Turkey

**Keywords:** Dilation, Corneal pachymetry, Lenses, intraocular, Anterior chamber, Children, Dilatação, Paquimetria corneana, Lentes intraoculares, Câmara anterior, Criança

## Abstract

**Purpose:**

Pharmacological pupillary dilation is performed in comprehensive ophthalmological examinations and before biometric measurements. So far, there is no consensus regarding its impact on biometric measurements. This study’s aim was to investigate the effects of pharmacological pupillary dilation on ocular biometric measurements in healthy children.

**Methods:**

This was a prospective, observational, non-randomized study of children (4-18 years of age) who were admitted for routine ophthalmological examination. Bio metric measurements were performed, using a non-contact optical biometry device, both before and after pharmacological pupillary dilation with cyclopentolate hydrochloride. Intraocular lens power calculations were performed using Hill-RBF, Barrett, Olsen, Sanders-Retzlaff-Kraff/Theoretical, Holladay, and Hoffer Q formulas. Descriptive statistical analyses were also performed. The Wilcoxon signed-rank test was used to compare measurements before and after pharmacological pupillary dilation. Relationships between variables were analyzed using the Spearman-Brown rank correlation coefficient.

**Results:**

The study included 116 eyes of 58 children (mean age, 8.4 ± 0.32 years; 34 girls). Significant changes were observed after pupillary dilation, compared with before pupillary dilation, in terms of anterior chamber depth, aqueous depth, and central corneal and lens thicknesses. No significant change was observed in axial length. Intraocular lens power calculations revealed no significant changes after pupillary dilation in most formulas except for the Olsen formula. The intraocular lens power was significantly inversely correlated with axial length and anterior chamber depth.

**Conclusions:**

Pharmacological pupillary dilation in children appeared to have no impact on axial length and intraocular lens power, but caused a significant increase in anterior chamber depth. The difference in anterior chamber depth measurements before and after pupillary dilation could be related to the optical biometry device model used. These outcomes should be considered in intraocular lens power calculations performed using anterior chamber depth parameters.

## Abstract (pt)

**Objetivo:**

A dilatação pupilar farmacológica é rea lizada em exames oftalmológicos abrangentes e antes das medições biométricas. Até o momento, não há consenso sobre seu impacto nas medições biométricas. O objetivo deste estudo foi investigar os efeitos da dilatação pupilar nas medidas biométricas oculares em crianças saudáveis.

**Métodos:**

Estudo prospectivo, observacional e não randomizado de crianças (4-18 anos) que foram admitidas para exame oftalmológico de rotina. As medidas biométricas foram realizadas usando um dispositivo de biometria óptica sem contato, antes e após a dilatação pupilar farmacológica com cloridrato de ciclopentolato. Os cálculos de potência das lentes intraoculares foram realizados utilizando as fórmulas de Hill-RBF, Barrett, Olsen, Sanders-Retzlaff-Kraff/ Teórica, Holladay e Hoffer Q. Análises estatísticas descritivas também foram realizadas. O teste dos postos sinalizados de Wilcoxon foi usado para comparar as medidas antes e após a dilatação pupilar farmacológica. As relações entre as va riáveis foram analisadas pelo coeficiente de correlação de Spearman-Brown.

**Resultados:**

O estudo incluiu 116 olhos de 58 crianças (idade média de 8,4 ± 0,32 anos; 34 meninas). Alterações significativas foram observadas após a dilatação pupilar, em termos de profundidade da câmara anterior, profundidade do humor aquoso e espessura central da córnea e do cristalino. Nenhuma mudança significativa ocorreu no comprimento axial. Os cálculos de potência da lente intraocular não revelaram alterações significativas após a dilatação pupilar na maioria das fórmulas, com exceção da fórmula Olsen. O poder da lente intraocular foi significativamente inversa correlacionada com o comprimento axial e a profundidade da câmara anterior.

**Conclusões:**

A dilatação pupilar farmacológica em crianças parece não ter impacto no comprimento axial e no poder da lente intraocular, mas causou um aumento significativo na profundidade da câmara anterior. A diferença nas medidas da profundidade da câmara anterior antes e após a dilatação pupilar pode estar relacionada ao modelo do dispositivo de biometria óptica utilizado. Tais resultados devem ser considerados nos cálculos de potência da lente intraocular realizados usando parâmetros de profundidade da câmara anterior.

## INTRODUCTION

Biometry and intraocular lens (IOL) power calculation are used to measure parameters that determine the eye’s refractive power, and performing these measurements accurately can enhance treatment success^(1,2)^. Accurate keratometric and biometric measurements, and accurate IOL power calculation formulas, are essential aspects of modern cataract surgery and refractive surgery, which must be performed to meet increased patient expectations and achieve target refractive outcomes^(3)^. Currently, the main biometric measurement devices are the IOLMaster (Carl Zeiss Meditec, Jena, Germany) and the Lenstar (Haag-Streit AG, Koeniz, Switzerland). These optical biometry devices have become widely used for accurate measurements of axial length because of their ease of use, rapid measurement, and contact-free approach^(3)^. Non-contact optical biometry devices are used also in pediatric patients. However, their use in very young children is limited due to lack of patient cooperation^(2)^.

Pharmacological pupillary dilation is performed in com prehensive ophthalmological examinations, particularly before biometric measurements. There is not yet a clear consensus regarding the impact of pharmacological pupillary dilation on biometric measurements. The present study aimed to investigate the effects of pharmacological pupillary dilation on ocular biometric measurements in healthy children. For this purpose, healthy children’s pupils were dilated with cyclopentolate hydrochloride eyedrops; biometric data were compared between measurements performed before and after pupillary dilation.

## METHODS

This study included children aged 4-18 years who underwent routine ophthalmological examinations at our clinic in the University of Health Sciences Diyarbakır Gazi Yaşargil Training and Research Hospital between June 2017 and June 2018. Children under the age of 4 years were excluded because they could not comply with the procedure for non-contact optical biometry assessment. Children aged 4-6 years were also excluded if they did not cooperate sufficiently to complete the procedure. In addition, children were excluded if they had inadequate pupillary dilation, a history of trauma or intraocular surgery, cataracts, and/or eye infections or cornea injuries. The study was approved by the Clinical Research Ethics Committee of the University of Health Sciences Diyarbakır Gazi Yaşargil Training and Research Hospital (date: 29.12.2017), and the study protocol complied with the tenets of the Helsinki Declaration of 1975, as revised in 2000. Informed consent was obtained from all patients and/or their parents for the study.

Biometric measurements were performed using a non-contact optical biometry device (Lenstar, Haag-Streit AG, Koeniz, Switzerland) before and after pupillary dilation with cyclopentolate hydrochloride. All children’s pupils were dilated by drops of 1% cyclopentolate hydrochloride, 3 times at 10-minute intervals. Non-contact biometric measurements of dilated pupils were taken after the pupillary reaction to light had disappeared^(4)^ and a pupillary diameter of 6 mm had been achieved.

The effects of pupillary dilation were assessed by comparing biometric measurements recorded before and after pupillary dilation. Ocular biometric parameters evaluated in this study included axial length (AL), central corneal thickness (CCT), aqueous depth (AD), anterior chamber depth (ACD), and lens thickness (LT). In addition, IOL power calculations were performed using 6 currently available formulas: Hill-RBF, Barrett, Olsen, Sanders-Retzlaff-Kraff/Theoretical (SRK/T), Holladay, and Hoffer Q. All measurements were performed by the same person using the same device (Lenstar, Haag-Streit AG). Biometric measurements were repeated three times in each child. The measurements were considered unreliable, and the relevant patient was not included in the study, if the following deviations occurred: >0.1 mm in AL, >0.25 D in K1 (horizontal meridian), or >0.25 D in K2 (vertical meridian).

### Statistical analysis

Data analysis was performed using IBM SPSS Statistics for Windows (version 23.0; IBM Corp., Armonk, NY, USA). Data normality was assessed using the Kolmogorov-Smirnov test. Descriptive statistics were expressed as number and percentage for categorical variables and as mean and standard deviation for numerical variables. Before-after comparisons were performed by the Wilcoxon signed-rank test for non-normally distributed variables. The Spearman-Brown rank correlation coefficient was used to assess relationships between non-normally distributed numerical variables. The level of statistical significance was set at p<0.05.

## RESULTS

The study included 58 children with a mean age of 8.4 ± 0.32 years, of whom 34 (58.6%) were girls and 24 (41.4%) were boys. Measurements were performed in both eyes of each child; thus, data from 116 eyes were evaluated. The biometric measurements, using the optical biometry Lenstar model and multi-formula lens power calculations for emmetropia, before and after pupillary dilation are shown in table 1.

**Table 1:** Results of biometric measurements using optical biometry Lenstar model and multiple intraocular lens power calculations for emmetropia before and after pupillary dilation

	Before dilation	After dilation	p-value^*^
	Mean ± SD	Mean + SD	
**AL**	22.72 ± 0.11	22.74 ± 0.11	0.077
**ACD**	3.49 ± 0.03	3.66 ± 0.02	**<0.001**
**AD**	2.92 ± 0.03	3.10 ± 0.02	**<0.001**
**CCT**	564.28 ± 3.11	566.01 ± 3.07	**0.005**
**LT**	3.60 ± 0.02	3.44 ± 0.01	**<0.001**
**IOL power**			
Hill-RBF	24.59 ± 0.39	24.42 ± 0.38	0.747
Barrett	24.16 ± 0.39	24.12 ± 0.39	0.086
Olsen	23.88 ± 0.35	24.14 ± 0.35	**<0.001**
SRK/T	23.86 ± 0.36	23.85 ± 0.36	0.088
Holladay	24.08 ± 0.39	24.03 ± 0.38	0.979
Hoffer Q	24.28 ± 0.41	24.22 ± 0.41	0.904

**Figure 1:**
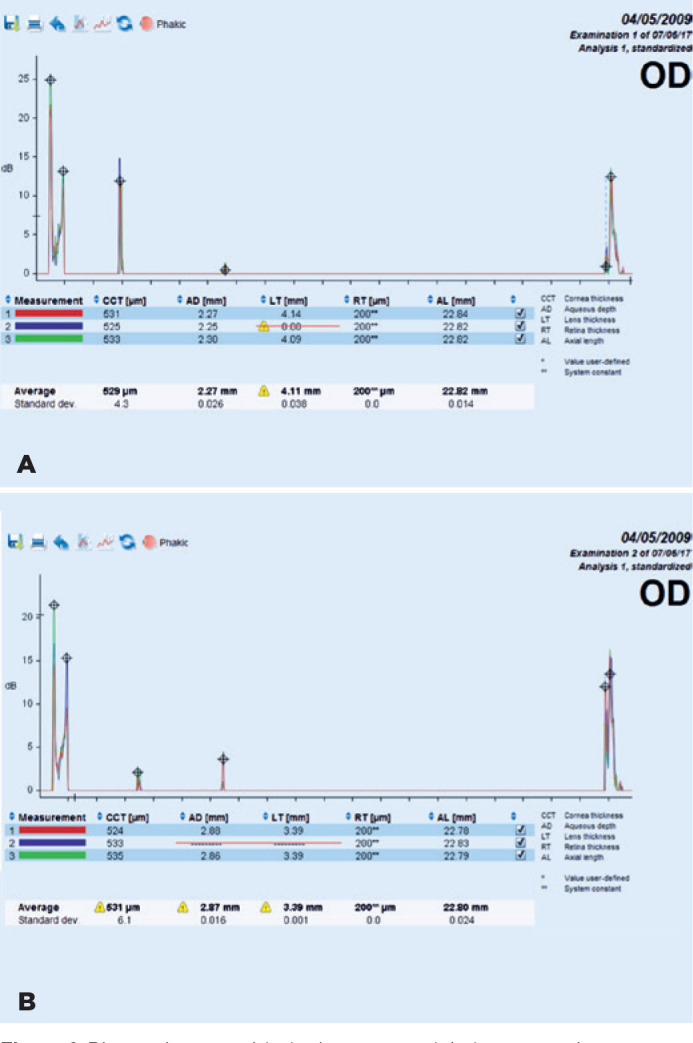
Biometric scan with the Lenstar model, demonstrating aqueous depth (AD) before (A) and after dilation (B) in a patient.

Significant changes were observed after pupillary dilation, compared to before pupillary dilation, in terms of ACD, AD, CCT, and LT values. A representative biometric scan by the Lenstar device, demonstrating the change in AD between before and after dilation in a patient, is presented in figure 1. No significant changes were observed in AL values; moreover, IOL power calculations revealed no significant changes after pupillary dilation in most formulas except for the Olsen formula.

Correlations between AL, determined using the optical biometry Lenstar model, and IOL power calculations for emmetropia, before and after pupillary dilation, are demonstrated in table 2. Significant strongly negative correlations were observed between AL and IOL power both before and after pupillary dilation, which indicated that IOL power decreased with increasing AL.

**Table 2:** Correlations between axial lengths determined using optical biometry Lenstar model and multiple intraocular lens power calculations for emmetropia before and after pupillary dilation

lOL power	Before dilation		After dilation	
	r	p-value^**^	r	p-value^**^
Hill-RBF	-0.896	**<0.001**	-0.885	**<0.001**
Barrett	-0.897	**<0.001**	-0.887	**<0.001**
Olsen	-0.798	**<0.001**	-0.796	**<0.001**
SRK/T	-0.918	**<0.001**	-0.912	**<0.001**
Holladay	-0.905	**<0.001**	-0.900	**<0.001**
Hoffer Q	-0.893	**<0.001**	-0.887	**<0.001**

IOL= intraocular lens; SRK/T= Sanders-Retzlaff-Kraff/Theoretical.

** Spearman-Brown rank correlation coefficient.

Correlations between ACD, determined using the optical biometry Lenstar model, and IOL power calcu lations for emmetropia, before and after pupillary dilation, are demonstrated in table 3. Significant moderately negative correlations were observed between ACD and IOL power, both before and after pupillary dilation, which indicated that IOL power decreased with increa sing ACD.

**Table 3:** Correlations between anterior chamber depths determined using optical biometry Lenstar model and multiple intraocular lens power calculations for emmetropia before and after pupillary dilation

lOL power	Before dilation		After dilation	
	r	p-value^**^	r	p-value^**^
Hill-RBF	-0.669	**<0.001**	-0.617	**<0.001**
Barrett	-0.645	**<0.001**	-0.624	**<0.001**
Olsen	-0.572	**<0.001**	-0.470	**<0.001**
SRK/T	-0.670	**<0.001**	-0.637	**<0.001**
Holladay	-0.673	**<0.001**	-0.631	**<0.001**
Hoffer Q	-0.674	**<0.001**	-0.631	**<0.001**

IOL= intraocular lens; SRK/T= Sanders-Retzlaff-Kraff/Theoretical.

** Spearman-Brown rank correlation coefficient

## DISCUSSION

Measurements of ocular biometric parameters by the partial coherence interferometry-based IOLMaster device are widely used and regarded as the gold standard. Lenstar, which was used in the present study, is an alternative biometric device; its measurements are based on the use of low coherence reflectometry. Lenstar allows non-contact measurement of the following parameters in a single set of scans: AL, ACD, CCT, LT, keratometry, retinal thickness, white-to-white distance (WTW), and eccentricity of the visual optical line^(5)^. A study of healthy volunteers demonstrated that IOLMaster and Lenstar measurements showed good agreement for many parameters and that agreement was excellent for AL^(5)^. Lenstar has demonstrated very good reproducibility for AL measurements and IOL power calculations^(6)^.

Various formulas are available for IOL power calculation. AL and ACD are among the important biometric parameters used in IOL power calculation^(7)^. Because formulas for IOL power calculation have been derived from studies performed on adult eyes, it is unclear which of these formulas is optimal for use in children^(8)^. IOL power prediction errors are reportedly highly variable in pediatric patients. O’Gallagher et al.^(9)^ compared four formulas (Hoffer Q, Holladay I, SRK-II, SRK/T) and concluded that SRK/T was more accurate than SRK-II in their pediatric patients; moreover, based on theoretical analysis, Hoffer Q was likely to be more accurate. In the present study, we used the following six formulas for IOL power calculation: Hill-RBF, Barrett, Olsen, SRK/T, Holladay, and Hoffer Q.

Studies evaluating the effects of pupillary dilation on biometric parameters and IOL power are typically conducted in adults, including among older patients with cataract. Adler et al.^(10)^ performed a study on 318 eyes of adults with cataract (age range, 20-95 years) and evaluated the effects of pupillary diameter on biometric measurements using IOLMaster. They reported that pupillary dilation via 0.5% tropicamide and 10% phenylephrine had no effects on AL or IOL power (SRK/T formula). Arriola-Villalobos et al.^(11)^ performed biometric measurements using Lenstar in 72 eyes, both before and after pupillary dilation with 1% tropicamide, in older patients undergoing cataract surgery; they reported a significant increase in ACD, but not in other biometric parameters, or in IOL power calculated by the Holladay II and SRK/T formulas^(11)^. In a similar study by the same group (Arriola-Villalobos et al.), measurements were performed on 81 eyes both before and after pupillary dilation with the IOLMaster 700 device (using swept source optical coherence tomography). They reported significant changes in ACD, CCT, LT, and WTW after pupillary dilation, but found no changes in IOL power calculated by the Holladay II and SRK/T formulas^(12)^. Bakbak et al.^(6)^ used Lenstar to perform biometric measurements in 33 eyes of adult patients with cataract, both before and after pupillary dilation with 1% tropicamide; they found a significant increase in ACD, but no changes in AL or IOL power. Rodriguez-Raton et al.^(13)^ evaluated 107 adult eyes with cataract using IOLMaster and found that pupillary dilation with 0.5% tropicamide and 0.5% phenylephrine hydrochloride led to a significant increase in ACD. They reported that no significant change was observed in AL; however, IOL power showed a significant change when calculated using the Haigis formula, but no change when calculated using the SRK/T formula^(13)^. Can et al.^(14)^ evaluated 72 eyes in a study group comprised of adult healthy volunteers and cataract patients using AL-Scan, both before and after pupillary dilation with 1% cyclopentolate hydrochloride. They reported that ACD increased after pupillary dilation, whereas AL and IOL power showed no significant differences.

The effects of pupillary dilation on biometric measurements have also been studied in healthy volunteers. Huang et al.^(15)^ evaluated the effects of pupillary dilation on biometric measurements and IOL power calculations, using two different devices (Lenstar LS900 and IOLMaster), in the left eyes of 43 healthy volunteers (age range, 18-37 years); they calculated IOL power using the SRK/T, Holladay 1, Hoffer Q, and Haigis formulas. The values obtained by each device exhibited good agreement with each other. Moreover, pupillary dilation with 0.5% tropicamide and 0.5% phenylephrine hydrochloride was found to increase the ACD and horizontal iris width (i.e., WTW), but did not affect AL or corneal curvature measurements. Khambhiphant et al.^(16)^ used IOLMaster to evaluate the effects of pupillary dilation in 384 eyes of healthy volunteers (age range, 21-79 years) and found no change in AL; however, they observed a significant increase in ACD after pupillary dilation. In addition, they calculated IOL power using the SRK/T formula and reported no significant change after pupillary dilation^(16)^. In a similar study by that group (Khambhiphant et al.), 373 eyes of healthy adults (age range, 18-93 years) were evaluated; they again found no significant change in AL, but observed a significant increase in ACD. In the second study, Khambhiphant et al. used the Haigis formula, rather than the SRK/T formula, and observed a significant change in IOL power^(17)^.

In the literature, there have been limited studies in volving ocular biometric measurements in pediatric patients. In a population-based study, the distribution of ocular biometric parameters was investigated in school-age children (age range, 5-8 years) and the AL and ACD were found to be normally distributed^(18)^. In another population-based study of school-age children (n=4870; age range, 6-12 years), IOL power was significantly inversely correlated with AL and ACD (r=-0.675 and r=-0.407, respectively)^(19)^; in that study, each 1-mm increase in AL reduced IOL power by 4.41 diopters^(19)^. Similarly, in the present study, IOL power was significantly inversely correlated with AL and ACD. The present study revealed a significant increase in ACD, but no change in AL after pupillary dilation in children. Moreover, IOL power was not influenced by pupillary dilation in most formulas except for the Olsen formula. The present study results were consistent with those of the above-mentioned studies, as well as with those of studies conducted in adults.

The present study was performed on healthy volunteer participants; accordingly, children under the age of 4 years were excluded because they could not comply with the approach necessary for non-contact optical biometry measurements. However, in cases where mea surements are necessary, such as planned surgery for children aged 2-4 years, using this device may be appropriate.

In conclusion, pharmacological pupillary dilation in children appeared to have no impact on AL values and IOL power calculations. However, a significant increase was observed in ACD values after pupillary dilation. The difference in ACD measurements before and after pupillary dilation could be related to the optical biometry device model used. These outcomes should be considered in IOL power calculations performed using ACD parameters.
